# Effects of a Short Physical Exercise Intervention on Patients with Multiple Sclerosis (MS)

**DOI:** 10.3390/ijms160715761

**Published:** 2015-07-10

**Authors:** Arno Kerling, Karin Keweloh, Uwe Tegtbur, Momme Kück, Lena Grams, Hauke Horstmann, Anja Windhagen

**Affiliations:** 1 Institute of Sports Medicine, Hannover Medical School, Carl-Neuberg-Str. 1, 30625 Hannover, Germany; E-Mails: sportmedizin@mh-hannover.de (K.K.); tegtbur.uwe@mh-hannover.de (U.T.); kueck.momme@mh-hannover.de (M.K.); sportmedizin-akk@mh-hannover.de (L.G.); sportmedizin-osp@mh-hannover.de (H.H.); 2 Department of Neurology, Hannover Medical School, 30625 Hannover, Germany; E-Mail: windhagen.anja@mh-hannover.de

**Keywords:** endurance training, resistance training, VO_2peak_, VAT, quality of life, MFIS, SF36

## Abstract

Background: The aim of this prospective randomized controlled trial was to investigate if a short-term endurance or combined endurance/resistance exercise program was sufficient to improve aerobic capacity and maximum force in adult patients (18–65 years) with multiple sclerosis (MS). Methods: All patients performed a three-month exercise program consisting of two training sessions per week, lasting 40 min each, with moderate intensity. All patients had a maximum value of 6 (low to moderate disability) on the Expanded Disability Status Scale (EDSS). One group (combined workout group (CWG); 15 females, 4 males) completed a combined endurance/resistance workout (20 min on a bicycle ergometer, followed by 20 min of resistance training), while the other group (endurance workout group (EWG); 13 females, 5 males) completed a 40 min endurance training program. Aerobic capacity was assessed as peak oxygen uptake, ventilatory anaerobic threshold, and workload expressed as Watts. Maximum force of knee and shoulder extensors and flexors was measured using isokinetic testing. Quality of life was assessed with the SF-36 questionnaire, and fatigue was measured using the Modified Fatigue Impact Scale. Results: Both training groups increased in aerobic capacity and maximum force. EWG, as well as CWG, showed improvement in several subscales of the SF-36 questionnaire and decrease of their fatigue. Conclusion: A short exercise intervention increased both aerobic capacity and maximum force independent of whether endurance or combined endurance/resistance workouts were performed.

## 1. Introduction

Multiple sclerosis (MS) is a chronic inflammatory autoimmune disease and is associated with reduced physical capacity and quality of life (Qol) [1,2]. Today, it is known that physical exercise does not lead to relapse or a faster progression of the disease but decreases fatigue and improves fitness, Qol [3,4,5,6,7,8], and walking ability in particular walking speed and endurance [9]. Despite these facts, patients with MS have been reported to undertake less sporting activity than the normal population [10], resulting in reduced physical capacity [11]. This sedentary lifestyle in patients with MS is mainly caused by deficits of the musculoskeletal system but also by psychosocial factors, such as loss of enjoyment of exercise, a lack of belief in the success of training or fear of relapse [10,12].

More than half of the patients with MS have been reported to suffer from heat sensitivity, which results in a reversible worsening of MS symptoms, for example, when participating in physical activities [13]. Since resistance training leads to a lesser increase in the core temperature than endurance training, it is better tolerated for heat-sensitive patients with MS [14].

In general, inadequate levels of physical fitness lead to higher cardiovascular and general mortality [15]. Additionally, deficient cardiopulmonary fitness is an important cardiovascular mortality risk factor and even more significant than classical risk factors, such as diabetes mellitus, overweight, smoking and hypercholesterolemia [16]. In this regard, it is necessary, especially for patients with MS and low physical fitness, to improve their physical fitness.

Wens I. *et al.* [17] found a smaller mean cross sectional area (CSA) of all muscle fibers as well as a smaller CSA of type I, II and IIa fibres of the quadriceps in 34 patients with MS, resulting in a lower muscle strength of the lower limb compared to healthy controls. A systematic training program can counteract this deconditioning caused by inactivity.

The World Health Organization recommends 150 min of moderate-intensity activity per week for healthy adults [18]. However, these recommendations are difficult to implement for patients with MS.

Physical limitation of patients with MS is mainly caused by decreased VO_2max_ and reduced muscle strength [1,19]. Therefore, in this prospective randomized trial, two different training regimes (an endurance and a combined endurance/resistance training program) were compared to investigate their effects on aerobic capacity and maximum force in patients with mild to moderate MS in short physical exercise units (two times a week, for forty minutes). Additionally, we evaluated the effects of the programs on fatigue and Qol as secondary outcomes. We hypothesized that the endurance program has more significant effects on aerobic capacity while the combined program has a better effect on maximum force.

## 2. Results

**Figure 1:**
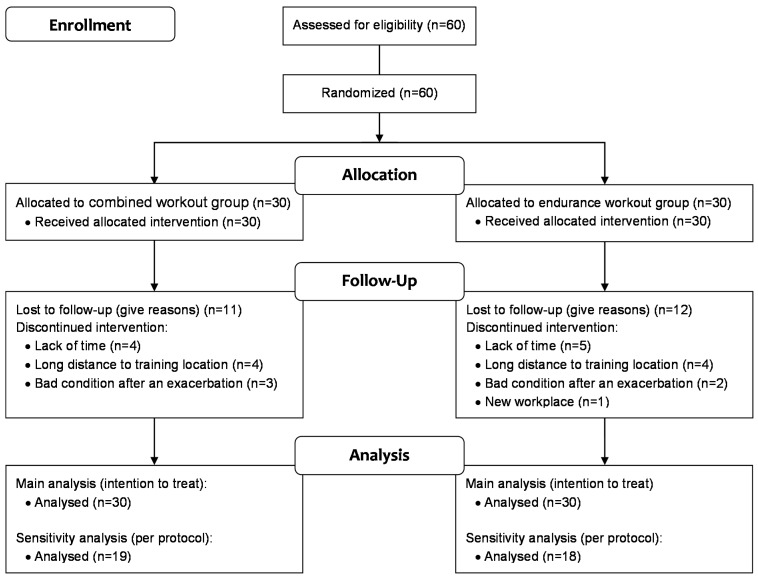
Flow chart of drop outs.

Between the baseline examination and the end of the training program, 18 patients dropped out because of personal reasons unrelated to the intervention (lack of time, new workplace, long distance to the location of the training). Five patients (8%) experienced an exacerbation of MS symptoms before completing the training program. Overall, 23 patients (38%) were excluded for the sensitivity analysis (Figure 1).

### 2.1. Aerobic Capacity

Both groups were comparable in age, BMI, sex, and intensity of the MS (Table 1). Additionally there was no significant difference between both groups concerning load (W) (*p =* 0.69), VO_2peak_ (mL/min/kg_BW_) (*p =* 0.85) and VAT (W) (*p =* 0.68) (Table 2).

**Figure 2:**
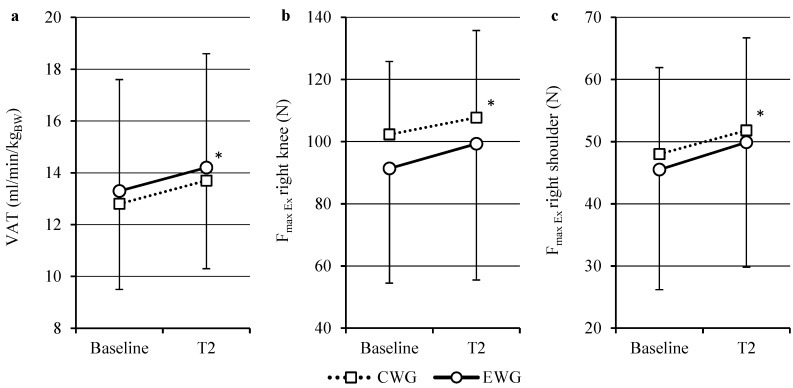
Ventilatory anaerobic threshold (**a**), maximum force for the right knee (**b**) and shoulder extensors (**c**) at baseline and after training (T2). * *p <* 0.01 over time in both groups with no differences between groups.

Aerobic capacity, as demonstrated by the parameters listed below, was significantly higher in both groups after training, but there was no significant difference between the two training types (Table 2). After training, both groups improved their parameters of physical capacity (Figure 2a); there was no significant time × group effect. Although, in the case of VO_2peak_, the main analysis showed no significant time effect, the sensitivity analysis revealed a significant improvement over time in both groups (VO_2peak_ in mL/min *p <* 0.01, η^2^ = 0.39 respectively in mL/min/kg_BW_ *p <* 0.01, η^2^ = 0.37) with no differences between groups (*p =* 0.96 respectively *p =* 0.72).

**Table 1:** Baseline characteristics.

Parameter	CWG (*n =* 30)	EWG (*n =* 30)	*p*-Value
Age (years)	42.3 ± 9.0	45.6 ± 11.4	0.21
Height (cm)	170 ± 5	169 ± 4	0.66
Body weight (kg)	71.4 ± 12.1	70.8 ± 11.9	0.84
BMI (kg/m^2^)	24.5 ± 3.6	24.7 ± 4.0	0.86
EDSS	2.6 ± 1.1	3.1 ± 1.3	0.09
MS specific medication	20/30	21/30	
Female/male	24/6	20/10	

Values are mean ± SD.

**Table 2:** Physical capacity parameters at baseline and after training (T2).

Parameter	CWG		EWG		*p*-Value		
	Baseline	T2	Baseline	T2	Time	η^2^	Time × Group
Load (W)	124 ± 48	131 ± 53	119 ± 44	124 ± 41	<0.01	0.17	0.78
Load (W/kg)	1.75 ± 0.61	1.84 ± 0.68	1.71 ± 0.66	1.78 ± 0.60	<0.01	0.17	0.78
Lactate_50W_ (mmol/min)	1.45 ± 0.51	1.31 ± 0.51	1.57 ± 0.79	1.35 ± 0.52	<0.01	0.14	0.54
Lactate_max_ (mmol/min)	5.43 ± 2.03	5.90 ± 1.97	4.80 ± 2.89	5.14 ± 2.50	<0.01	0.12	0.66
Heart rate_rest_ (bpm)	92 ± 12	90 ± 11	88 ± 12	85 ± 13	0.02	0.09	0.63
Heart rate_50W_ (bpm)	120 ± 15	115 ± 15	116 ± 15	110 ± 14	<0.01	0.31	0.74
Heart rate_max_ (bpm)	161 ± 17	162 ± 18	152 ± 24	152 ± 24	0.53	–	0.85
VO_2peak_ (mL/min)	1684 ± 601	1756 ± 599	1632 ± 539	1676 ± 494	0.12	–	0.71
VO_2peak_ (mL/min/kg_BW_)	23.8 ± 7.8	24.6 ± 7.4	23.5 ± 8.2	23.7 ± 7.1	0.24	–	0.72
Borg scale	16.5 ± 1.4	16.0 ± 1.9	16.3 ± 1.4	15.7 ± 1,70	0.02	0.09	0.68
VAT (W)	51.0 ± 23.4	55.8 ± 24.8	50.5 ± 23.1	57.6 ± 25.5	<0.01	0.26	0.39
VAT (mL/min/kg_BW_)	12.8 ± 3.3	13.7 ± 3.4	13.3 ± 4.3	14.2 ± 4.4	<0.01	0.35	0.79

Values are mean ± SD; VO_2peak_, peak oxygen uptake; BW, body weight; VAT, ventilatory anaerobic threshold.

The spiroergometric tests were stopped by the patient with the onset of subjective exertion with an average value of 16 on the Borg scale, with no significant differences in maximal heart rate in both groups over time and between the two training types (Table 2). Additionally there was no significant change in the EDSS over time in both groups (CWG: 2.6 + 1.1, 2.6 + 1.1; EWG: 3.1 + 1.3, 3.1 + 1.3; *p =* 0.16).

### 2.2. Isokinetics

Measurements of *F*_max_ for the knee flexors (FL, hamstrings) and extensors (EX, quadriceps femoris), as well as for the shoulder extensors and flexors, showed significantly higher results for both the left- and right-hand side after the training period (Figure 2b,c) with the exception of *F*_max Ex_ left (Table 3). Again, there was no significant difference between the types of training. The sensitivity analysis of F_max Ex_ of the left knee showed significantly higher values after training (*p =* 0.01, η^2^ = 0.18) with no differences between groups (*p =* 0.82).

**Table 3:** Isokinetic parameters at baseline and after training (T2).

Parameter	CWG		EWG		*p*-Value		
	Baseline	T2	Baseline	T2	Time	η^2^	Time × Group
Knee							
*F*_max Ex_ right (N)	102.3 ± 23.5	107.7 ± 28.0	91.4 ± 36.9	99.3 ± 42.3	<0.01	0.15	0.50
*F*_max Ex_ left (N)	105.5 ± 28.1	108.2 ± 33.1	92.7 ± 39.3	95.6 ± 43.8	0.23	-	0.95
*F*_max Fl_ right (N)	55.3 ± 16.0	61.3 ± 18.7	51.0 ± 21.0	55.9 ± 24.6	0.01	0.18	0.72
*F*_max Fl_ left (N)	58.2 ± 20.2	64.0 ± 23.7	48.7 ± 23.5	51.7 ± 24.85	<0.01	0.16	0.31
Shoulder							
*F*_max Ex_ right (N)	48.0 ± 13.9	51.8 ± 14.9	45.5 ± 19.3	49.9 ± 20.1	<0.01	0.14	0.85
*F*_max Ex_ left (N)	46.3 ± 17.5	50.0 ± 18.9	43.3 ± 17.3	46.9 ± 18.6	<0.01	0.19	0.98
*F*_max Fl_ right (N)	34.2 ± 9.6	36.5 ± 10.0	35.3 ± 12.6	36.9 ± 14.1	0.02	0.10	0.67
*F*_max Fl_ left (N)	35.8 ± 13.9	36.9 ± 12.4	34.0 ± 12.1	35.9 ± 12.5	0.04	0.07	0.60

Values are mean ± SD. Ex, extensor; Fl, flexor.

### 2.3. Questionnaires

#### 2.3.1. SF-36

Both groups showed significantly better results for the subscales 2, 4, 5, 6 and 8 and for the mental health sum score at the end of the study (Table 4).

#### 2.3.2. MFIS

In both groups, we found a significant reduction of the fatigue score for all patients (Table 4) as well as for the patients with a pathological score over 38 (*n =* 17; 52 ± 8 before training, 42 ± 11 after training; *p <* 0.001).

### 2.4. Training Program

Overall more than 90% of the training sessions (on average 24/26) were completed.

**Table 4:** Questionnaire parameters at baseline and after training (T2).

Parameter	CWG		EWG		*p*-Value		
	Baseline	T2	Baseline	T2	Time	η^2^	Time × Group
MFIS score	35.5 ± 17.0	30.6 ± 16.7	35.1 ± 17.4	30.3 ± 18.1	<0.01	0.24	0.97
SF 36							
Scale 1 (physical functioning)	71.7 ± 21.3	71.5 ± 22.9	60.7 ± 27.1	62.7 ± 26.6	0.43	–	0.33
Scale 2 (role limitations due to physical limitations)	50.0 ± 44.9	62.5 ± 42.3	42.1 ± 47.2	50.0 ± 42.0	0.03	0.10	0.63
Scale 3 (bodily pain)	87.9 ± 19.6	87.4 ± 17.4	71.3 ± 24.4	72.4 ± 23.7	0.90	–	0.67
Scale 4 (general health perceptions)	46.9 ± 19.3	49.6 ± 22.4	43.6 ± 24.0	48.8 ± 25.6	0.03	0.10	0.48
Scale 5 (vitality)	47.5 ± 18.7	49.0 ± 20.4	44.8 ± 23.8	50.7 ± 22.3	<0.01	0.18	0.07
Scale 6 (social functioning)	72.9 ± 28.9	76.6 ± 24.3	68.2 ± 30.6	80.1 ± 24.3	<0.01	0.21	0.07
Scale 7 (role limitations caused by emotional problems)	66.7 ± 48.2	65.3 ± 52.5	78.8 ± 40.6	90.9 ± 25.6	0.36	–	0.25
Scale 8 (mental health)	65.8 ± 20.7	67.2 ± 19.1	62.0 ± 20.1	67.8 ± 18.0	<0.01	0.17	0.07
Physical health	44.7 ± 9.1	46.2 ± 9.1	39.0 ± 10.8	39.6 ± 11.3	0.16	–	0.56
Mental health	44.9 ± 13.6	45.4 ± 13.4	46.7 ± 11.7	51.4 ± 8.6	0.04	0.09	0.01 (η^2^ = 0.13)

Values are mean ± SD.

## 3. Discussion

Our results suggest that in patients with MS, regular training for 80 min per week, at a moderate intensity, increases aerobic capacity and maximum force-against our hypothesis-independent of the type of training.

### 3.1. Aerobic Capacity

The subjective perceived exertion measured with the Borg scale was, on average, 16 (between hard and very hard) for both groups, which indicated that cardiopulmonary exertion was not achieved in all patients. This finding is in line with Heine *et al.* [20], who found that only 23% of their patients with low to moderate MS achieved an exertion of 18 or greater on the Borg scale. Nevertheless, our patients improved their endurance capacity both in VO_2peak_ (although the main analysis showed no significant time effect, the sensitivity analysis revealed a significant improvement over time) and VAT, so a motivation-dependent effect seems unlikely. Even taking into account day-to-day variation in patients with MS, our results of an improvement of approximately 10% in VO_2peak_ (in the sensitivity analysis) can be interpreted as a real training effect [21].

A better endurance capacity after the training period was apparent from a lower heart rate at rest and at 50 W and lower lactate values at 50 W in both groups. These results can be explained by a right shift of the lactate performance curve [22]. Baseline levels of VO_2peak_ from all our patients (22 ± 7 mL/min/kg_BW_) were reduced, compared to healthy persons and are comparable with other studies taking into account the intensity of MS measured with EDSS [1,2,3]. Interestingly, both training groups increased their aerobic capacity, although the CWG group only performed 40 min per week of aerobic training on a bicycle ergometer.

Mostert and Kesselring [1] found an average increase in oxygen uptake of 12% at the aerobic threshold for 26 patients with MS cycling five times per week, for 30 min, over 3–4 weeks at the VAT under aerobic conditions. However, VO_2peak_ in this patient group was not improved by exercise therapy. Our results showed a significant growth of VO_2_ at the VAT and at least in the sensitivity analysis of VO_2peak_; one possible explanation is that our patients performed their training at a higher intensity in the aerobic-anaerobic transition area. Although higher lactate values can also be a sign of increased motivation or volitional exhaustion at a later time point, the Borg scale was not different after the incremental tests before and after training for both groups. In addition, motivation-independent parameters, such as VAT, lactate at 50 W, and heart rate at 50 W, showed a significant improvement of aerobic capacity. Compared with other endurance training studies in patients with MS, the improvement of VO_2peak_ in our study was less pronounced [2,3]. Patients in a study by Bjarnadottir *et al.* [2] showed an increase in endurance capacity (15% in VO_2peak_ and 18% increase in VAT) after training three times a week for five weeks on a bicycle ergometer. After a training session performed three times a week, for 40 min, on a combined arm and leg ergometer, Petajan *et al.* [3] observed a 22% increase in VO_2max_ after 15 weeks. The lower results in our patients could be explained by the lower frequency or the shorter duration of our training sessions. However, our results suggest that even 40 min of aerobic training per week (in combination with 40 min resistance training) may be enough for poorly-trained persons to improve their aerobic capacity significantly. Although Motl *et al.* [23] reported significant improvements in walking mobility after eight weeks of combined training, to our knowledge we are the first group who describes a benefit on aerobic capacity in a combined exercise program measured with spiroergometric parameters. This is contrary to the results of Romberg *et al.* [24] who found no significant change in aerobic capacity after a 26 week, home-based, combined training.

### 3.2. Muscle Strength

Surprisingly, participants in both of our training groups enhanced their maximum force for shoulder and knee extensors and flexors with no significant group effect, although the EWG group performed only endurance training. However, some of the participants in the EWG group also used a cross-trainer, a rowing ergometer or an arm ergometer, besides cycling ergometry, so shoulder and knee muscles were trained regularly. Therefore, these patients could have enhanced their results in isokinetic testing. This is in line with the study from Petajan *et al.* [3] who also found an improvement in muscle strength of the upper and lower extremity in a sole endurance training regime.

A recent study from Wens *et al.* [25] showed a significant improvement of a 24-week combined exercise program on muscle strength of the knee extensors and flexors emphasized in the hamstrings. Other combined training studies observed no, or only a modest, effect on muscle strength of quadriceps and hamstrings.

### 3.3. Quality of Life

Examination of Qol in patients in a study by Bjarnadottir *et al.* [2], which was determined with the SF-36 questionnaire, showed a tendency towards an improvement in five of eight subscales and was significant for subscale 5 (vitality). Mostert and Kesselring’s [1] study also showed a significant increase in subscales 5 and 6 (vitality and social function). Our results were in line with these studies and showed a significant improvement in both groups for subscales 2 (role limitations due to physical limitations), 4 (general health perceptions), 5 (vitality), 6 (social functioning) and 8 (mental health), and for the mental health sum score. In addition to training effects, improvement of psychological subscales could be explained by social interaction and social support from peers and therapists. No measurable effect on the physical sum score was seen in the EWG group or in the CWG. This is contrary to the results from Dalgas *et al.* [26], who performed a 12-week progressive resistance training program for patients with MS and a 12-week follow up trial (after twelve weeks, the exercise group continued training without supervision and the control group was offered the same intervention as the exercise group). They found a significant increase in the knee extensor strength and functional capacity score of the lower extremities in both groups after training. A significant increase in the physical sum score and a trend for the mental component of the SF-36 were seen for the exercise group and for the mental sum score in the control group after exercise. Referring to our patients the EDSS average was 3.0 ± 1.3 (in the sensitivity analysis) compared with 3.7 ± 0.9 in the study by Dalgas *et al.* [26]; thus, we assume that there was no effect since our patients were already in a better condition at baseline and a training effect in the physical sum score could only be seen in the patients with a greater level of disability.

### 3.4. Fatigue

The effect of exercise training on fatigue is inconsistent [6]; some studies performing endurance [27,28], resistance [26] or combined training [29] showed a significant improvement, while others did not [1,3,30]. In some of the studies, not all patients suffered from fatigue, which was also the case in our study. Nonetheless, in our study, for both the patients suffering from fatigue (MFIS > 38) and for the whole group, we found a significant improvement in fatigue, as determined using MFIS (Table 4).

### 3.5. EDSS

Golzari *et al.* [31] found a significant decrease in the EDSS after eight weeks of training (from 2.1 to 1.7) in women. In that study, IL-17 and IFN-y production also decreased, and they explained the clinical improvement with training-induced anti-inflammatory effects. In contrast, EDSS was stable in both of our groups, which is in accordance with other studies [2,3] which, likewise, showed no significant effect of exercise training on EDSS. A review on this question published by Dalgas and Stenager [32] stated that it is not clearly established if exercise in patients with MS has a disease-modifying effect or not, although there are individual studies indicating this.

### 3.6. Dropout

Five patients (8%) could not complete the training because of a relapse, taking into account the existing literature we are sure this was not caused by our training intervention. Petajan *et al.* [3] (13% experienced an exacerbation of MS symptoms with similar frequency in the exercise and non-exercise group) (19) and Bjarnadottir *et al.* [2] (dropout rate of 9% and 8% in the exercise and the control groups, respectively, because of a relapse before starting the training) reported a similar dropout rate in their exercise as well as in their non-exercise groups The other dropouts were caused by circumstances unrelated to the intervention (lack of time *n =* 9, to long distance to training location *n =* 8 new workplace *n =* 1, Figure 1).

## 4. Patients and Methods

### 4.1. Patients and Study Design

The study initially involved 60 patients (44 females, 16 males), who were recruited directly through the MS Healthcare Center of the Hannover Medical School by practicing neurologists in the region of Hannover, and through the newsletter of the local MS society. The inclusion criteria for participation in the study were diagnosed MS, adult age (18–65 years), and mobility with a maximum value of 6 (low to moderate disability) on the Expanded Disability Status Scale (EDSS). Inclusion in the study was not influenced by MS specific medication (e.g., Glatirameracetat, Interferon Natalizumab). Reasons for exclusion were additional cardiovascular and orthopaedic diseases, pregnancy and regular physical training over the previous 12-month period. The patients were randomized after an initial examination by age, sex, Body Mass Index (BMI) and EDSS into either the combined workout group (CWG) or the endurance workout group (EWG). Spiroergometry, isokinetics, a neurological examination, and completion of the questionnaires were performed at baseline examination and after completing the training program after three months. The allocation was concealed to all researchers conducting the second examination.

The study was approved by the ethics committee of the Hannover Medical School (Approval No. 3491, 2006). All participants were informed about possible risks and submitted their written consent before inclusion in the study.

### 4.2. Neurological Examination

#### 4.2.1. EDSS

The disease-specific degree of impairment was assessed using the EDSS, which evaluates the impairment in a variety of functional systems from a comprehensive neurological examination. Participants are scored on a scale ranging from 0 to 10 [33].

#### 4.2.2. Aerobic Capacity/Spiroergometry

Cardiopulmonary exercise testing is a valid method of measuring aerobic capacity in patients with mild to moderate MS [20]. For testing peak oxygen uptake (VO_2peak_), participants performed an incremental exercise test under supervision of a physician using a spirometric system (Oxycon Delta, CareFusion, Würzburg, Germany) on a speed-independent bicycle ergometer (Ergometrics 900s, Ergoline, Bitz, Germany) with 60 to 70 revolutions per minute, under electrocardiogram (ECG)-monitoring. The incremental test started with a load of 20 W, and the load increased 10 W every minute until the onset of subjective overexertion (peripheral muscle fatigue and/or dyspnoea). The subjective perceived exertion was assessed by the Borg scale ranging from extremely light to extremely hard [34]. The same test protocol was used after the training period.

Maximum oxygen uptake (VO_2max_) is an important criterion for endurance capacity and describes the maximum volume of oxygen the body can utilize per minute, under maximum load conditions. VO_2max_ is dependent on oxygen exchange, transport, and utilization systems [35]. In fact, VO_2max_ is often achieved only by competitive athletes or highly motivated subjects, and therefore, we have used the term VO_2peak_. Heart rate and oxygen uptake were continuously measured breath by breath and averaged over 10 s intervals. Blood pressure and blood lactate concentration were acquired at rest, 1 min after the start of testing and every 3 min during the test. Capillary blood samples of 20 µL were taken from the arterialized earlobe, deproteinized and then measured with a lactate analyzer (Ebio 6666, Eppendorf, Berlin, Germany). As a marker of oxidative muscle function, the anaerobic lactate threshold intensity was determined by the method of Roecker *et al.* [36].

The ventilatory anaerobic threshold (VAT) describes the transition from aerobic to partially anaerobic glucose metabolism in muscle. This transition results in increased carbon dioxide exhalation in comparison to oxygen uptake; the increasing build-up of lactate is buffered by bicarbonate and exhaled as carbon dioxide. The VAT represents the lower limit of the aerobic-anaerobic transition zone, is independent of motivation, and is an important parameter for training control. VAT was determined by the v-slope method published by Wassermann [37].

### 4.3. Isokinetics

Maximum strength was measured in the concentric mode. Isokinetic testing was performed by an experienced sports scientist approximately one hour after spiroergometry. All concentric torque values were done with the CON-TREX Multi-Joint System (CMV AG, Dübendorf, Switzerland) in the concentric/concentric mode. For shoulder and knee tests subjects were seated in an upright position of 85° flexion in the hip joint and 90° flexion in the knee joint. Seat belts were fastened. In order to get maximum stability of the tested lower limb a Velcro strap was fixed to the thigh. Subjects were positioned according to the manufacturer’s recommendations. Shoulder tests were performed with the center of rotation of the lever arm in extension to the center of rotation of the knee. This way a boxing-motion was accomplished. The dimension of the arm defined the range of motion. The range of motion for the knee tests was between 90° and 10° flexion. Testing involved a cycle of movements of a body segment at a constant velocity (60° per second), set at the start of each movement. The two antagonist muscle groups were activated alternatively with a loading level set by the patient. We measured the maximum force (*F*_max_) of the knee and shoulder extensors and flexors five times for five repetitions, with one-minute breaks between the repetitions. The highest value for each body segment was used for analysis.

### 4.4. Questionnaires

#### 4.4.1. Short Form-36 Health Survey (SF-36)

The SF-36 represents an established self-assessment method for evaluating Qol, which is widely used in clinical studies [38]. It consists of 36 individual items covering eight subscales of both physical health and mental health. The results are scaled between 0 and 100, with higher values representing a higher subjective Qol.

#### 4.4.2. MFIS (Modified Fatigue Impact Scale)

The MFIS is a shortened version of the Fatigue Impact Scale [39]. This questionnaire examines the impact of fatigue on physical, cognitive and psychosocial health. The score ranges between 0 and 84, where higher scores indicate a greater impairment of the patients. A score of 38 or greater is defined as pathological [40].

### 4.5. Training Program

The physician-supervised training program lasted three months and consisted of two training sessions per week, each of which was 40 min long and at moderate intensity. Training took place at the Institute of Sports Medicine of the Hannover Medical School. Both the combined training and endurance training programs started with a 20-min workout phase on a bicycle ergometer (Ergometrics 900s, Ergoline, Bitz, Germany) with 60 to 70 revolutions per minute. Heart rate was measured continuously via ECG, whereas blood pressure was measured every 5 min during the first workout phase. To achieve moderate intensity, participants performed at 50% of the maximum workload achieved during the incremental exercise test. At this intensity, all patients trained in the aerobic-anaerobic transition zone (above the VAT and below the anaerobic lactate threshold). Subjective perceived exertion on the Borg scale should be 13 at maximum. During the whole training program, the workload was adjusted according to the heart rate during the first training; the workload was increased by 10% when heart rate and exertion on the Borg scale decreased by a predetermined amount and blood pressure did not exceed 180/100 mmHg.

The second workout phase was performed directly after cycling. The endurance training could be continued on a cross-trainer (Motion Cross 500med; Emotion Fitness, Hochspeyer, Germany), a stepper (Motion Stair 500med; Emotion Fitness, Hochspeyer, Germany), an arm ergometer (Motion Body 500med; Emotion Fitness, Hochspeyer, Germany), a treadmill (Quasar; HP Cosmos, Nussdorf-Traunstein, Germany), a recumbent ergometer (Motion Relax 500med; Emotion Fitness, Hochspeyer, Germany) or a rowing ergometer (Concept2; Indoor Rower, Hamburg, Germany), as preferred by the participant. Heart rate was continuously monitored via ECG. The training heart rate was allowed to be a maximum of 10% above the average heart rate on the bicycle ergometer for all devices except for the recumbent ergometer (same heart rate) and the arm ergometer (heart rate should be approximately 10% lower). The intensity was adjusted according to the heart rate as mentioned above.

The patients in the CWG group underwent a dynamic resistance training program supervised by an experienced sports scientist, so they were able to perform two sets with 10 to 15 repetitions on each machine in a circuit; after completing 15 repetitions two times in a row, the resistance was intensified. As in aerobic training, subjective perceived exertion on the Borg scale should be 13 at maximum. Six out of eight strength machines (Cybex Eagle Line, Medway, MA, USA) could be used to achieve a complex, full-body workout in which multiple muscle groups were trained (leg press, hamstring curl, chest press, row, pull down, overhead press, abdominal, and back extension).

Both training regimes were well tolerated, and there was no worsening of symptoms as a result of the training sessions.

### 4.6. Statistics

As the main primary analysis, an “intention to treat” analysis was performed with the “last observation carried forward” principle for missing values. To test if this principle was too conservative, a sensitivity “per protocol” analysis was additionally undertaken. If not stated otherwise all shown data are the results of the main analysis.

All data are given as the mean ± standard deviation. Data were tested for a normal distribution using the Kolmogorov–Smirnov test. To establish the possible influence of the training program, analyses of variance with repeated measurements were performed before and after training, including the factor group CWG/EWG. To estimate the effect size, partial eta-squared (η^2^) was determined. Thereby, a η^2^ of 0.03 represented a power of 75% in the main analysis, 0.05 a power of 93% and 0.10 a power of 99%. For comparing characteristics of the CWG and EWG groups before training, unpaired, two-sided Student’s *t*-tests were performed, and Hedges g was calculated as the effect size. Significance was accepted at *p <* 0.05. All tests were performed with SPSS version 22 (IBM Corp., Armonk, NY, USA). Training results were included in the sensitivity analysis if more than 2/3 of the training sessions were attended.

## 5. Conclusions and Limitations

Regular training for 40 min, two times per week, with moderate intensity increases aerobic capacity and maximum force in patients with low to moderate MS independent of whether endurance or a combined type of training is used. Thus, we conclude that in patients with MS already, 40 min of endurance training are sufficient to improve aerobic capacity. If resistance training is not possible, *F*_max_ of the extremities can be enhanced when different types of endurance machines which specifically target the upper or lower limb (e.g., rowing, crosstrainer, arm ergometer) are used. Additionally, training improves Qol and reduces fatigue. Referring to the activity guidelines (aerobic training two times a week, for 30 min; strength training two times per week) [8], combined training should be done, preferably, but if not possible, endurance training is a good alternative in patients with mild to moderate MS.

The study was designed as presented above with two intervention groups without a control group, so training-specific effects cannot clearly be differentiated from intervention-bound effects. As the study involved patients whose participation was, in part, self-motivated, the results may not simply be applied to all patients with MS, as it may be assumed that patients interested in sports will show greater capacity and motivation. Improvements in subjective measures, such as Qol, can be explained by social or group effects independent of physical exercise. The patients were randomized as described above, and although there was no statistically significant difference between the two groups, the CWG group had significantly better results in the isokinetic testing of the knee extensors (*p =* 0.01) and the left flexors (*p =* 0.02) before starting the training.
